# Age Estimation of the Cervical Vertebrae Region Using Deep Learning

**DOI:** 10.3390/bioengineering13010007

**Published:** 2025-12-22

**Authors:** Zhiyong Zhang, Ningtao Liu, Ziyi Hu, Zhang Guo, Wenfan Jin, Chunxia Yan

**Affiliations:** 1 Key Laboratory of Shaanxi Province for Craniofacial Precision Medicine Research, College of Stomatology, Xi’an Jiaotong University, Xi’an 710004, China; 2 Department of Orthodontics, Affiliated Stomatological Hospital of Xi’an Jiaotong University, Xi’an 710004, China; 3 School of Computer, Luoyang Institute of Science and Technology, Luoyang 471023, China; 4 Key Laboratory of Intelligent Perception and Image Understanding of Ministry of Education, School of Artificial Intelligence, Xidian University, Xi’an 710071, China; 5 Department of Radiology, Affiliated Stomatological Hospital of Xi’an Jiaotong University, Xi’an 710004, China; 6 College of Forensic Medicine, Xi’an Jiaotong University, Xi’an 710061, China

**Keywords:** age estimation, cervical vertebrae region, deep learning, lateral cephalometric radiograph, segmentation mask

## Abstract

Since skeletal development is largely completed by adulthood, it is difficult for traditional methods to capture subtle age-related structural changes in bones and surrounding tissues. Recent advances in deep learning have demonstrated remarkable potential in medical image-based age estimation. The cervical vertebrae, as captured in lateral cephalometric radiographs (LCR), have shown particular value in such tasks. To systematically investigate the contribution of different vertebral representations to age estimation, we developed four distinct input modes: (1) Contour (C); (2) Mask (M); (3) Cervical Vertebrae (CV) and (4) Cervical vertebrae region (SR). Using a large-scale LCR dataset of 20,174 subjects aged 4–40 years, grouped into 5-year intervals, we evaluated these modes with deep learning models. The Mean Absolute Error (MAE) was used to evaluate performance. Results indicated that the SR mode achieved the lowest overall MAE, particularly for the C1–C4 combination, followed by CV, while C and M modes showed similar and poorer performance. For subjects younger than 25 years, MAEs for individual vertebrae (C1–2, C3, C4) were less than 5 years across all modes; however, in the 26–40 years group, MAEs for C and M modes exceeded 10 years, whereas CV and SR modes remained below 10 years for most combinations. Combining vertebrae consistently improved accuracy over individual ones, with continuous combinations (e.g., C1–2 + C3) outperforming discontinuous ones (e.g., C1–2 + C4). Visualization of age-related salience revealed that salient regions varied by input mode and expanded with increased information content. These findings underscore the critical importance of incorporating peripheral soft tissue and comprehensive vertebral context for accurate age estimation across a wide age spectrum.

## 1. Introduction

Forensic age estimation serves as a critical tool across a broad spectrum of legal, criminal, and humanitarian applications. It supports judicial processes in criminal cases, assists in civil matters such as immigration and adoption eligibility, and aids in humanitarian efforts, including refugee status determination and identification of unknown individuals. This multidisciplinary process typically integrates medical history reviews, physical examinations, and radiological assessments of skeletal and dental development [1,2,3,4,5]. Commonly examined anatomical regions include the hands, wrists, medial clavicle, and dentition, each providing developmental indicators at different stages of maturation [6,7,8,9,10,11]. Specifically, pelvic morphological changes, such as those of the auricular surface, pubic symphysis, and acetabulum, are among the most frequently utilized markers for adult age estimation [12]. However, methods based on acetabular morphological changes have been shown to systematically underestimate age in middle-aged and older individuals, with additional concerns regarding low inter-observer reliability [13,14].

Recent studies have highlighted the potential of cervical vertebrae-based age estimation using lateral cephalometric radiographs (LCRs) as an alternative to hand-wrist radiography. Advanced techniques, such as deep learning approaches focusing on cervical vertebrae segmentation, have yielded highly accurate bone age estimations from LCRs. The use of LCRs for age estimation offers advantages such as reduced radiation exposure and the ability to capture both ossification status and soft tissue information, making it a promising and reliable method for skeletal age assessment in orthodontic practice [15].

Although previous studies established the feasibility of cervical morphological measurements [16]—using width-to-height ratios of C3–C4 and morphological parameters such as concavity, anterior height, and angle of C2–C4 to construct regression models for bone age estimation [17,18]—these conventional methods relied on handcrafted feature extraction and linear modeling, thus failing to capture the complex nonlinear characteristics inherent in degenerative changes. Furthermore, due to the anatomical complexity of the vertebral arches and spinous processes, most earlier studies were limited to the more accessible vertebral bodies, neglecting other informative structures [19,20].

Researchers commonly classify cervical vertebrae into six developmental stages based on morphological changes to identify adolescent growth peaks. Parameters such as vertebral height-to-length ratios, curvature, and angles are also widely used for estimating adolescent age [17,18,21,22]. These assessments offer critical insights into skeletal development, particularly during adolescence, facilitating the prediction of mandibular growth peaks and their correlation with age [23,24,25]. In forensic contexts, especially for adults, degenerative changes and maturation degree of the cervical vertebrae can serve as important indicators of chronological age. However, traditional staging methods are highly subjective and heavily dependent on examiners’ clinical experience. Moreover, methodological constraints hinder the comprehensive characterization of age-related morphological changes in cervical vertebrae. Since cervical vertebral growth is generally completed by age 25, subsequent degenerative alterations—such as those affecting intervertebral discs, spinal curvature, and bone density [26,27,28,29,30,31]—are poorly captured by conventional metric approaches, limiting their utility in adult age estimation.

The limitations of these manual, feature-dependent methods highlight the need for more sophisticated analytical frameworks. Here, artificial intelligence (AI) has demonstrated transformative potential across diverse healthcare domains by enabling robust analysis of complex, multifactorial data for diagnostic and predictive tasks. For instance, in endocrinology, systematic reviews have shown that AI techniques (including machine learning and deep learning) can significantly enhance the diagnosis and prediction of outcomes for polycystic ovary syndrome (PCOS), a heterogeneous condition often challenging to diagnose with traditional criteria [32]. Similarly, in metabolic disease research, advanced gradient-boosting models like XGBoost, combined with explainable AI (XAI) methods, have achieved highly accurate and interpretable risk prediction for type 2 diabetes [33]. These applications demonstrate AI’s core strength in discovering subtle, non-linear relationships within high-dimensional data. Motivated by this proven capability, we apply deep learning to the specific challenge of cervical vertebrae-based age estimation. The goal is to leverage AI’s power to move beyond linear modeling and manually defined features, thereby capturing the intricate and combined age-related signals from both osseous structures and surrounding soft tissues in lateral cephalometric radiographs.

In recent years, deep learning models have excelled in various medical imaging tasks, such as object detection [34], registration [35], lesion segmentation [36], and classification [37]. These models offer significant advantages over traditional manual methods in terms of cost, time, automation, and objectivity. Deep learning is now being used to research cervical vertebral development stages and age estimation [38,39,40,41]. Recent studies have demonstrated the effectiveness of deep learning approaches, particularly convolutional neural networks (CNNs), in analyzing cervical vertebral maturation (CVM) and age-related changes in LCRs. Zhang et al. proposed an aging-related dynamic attention method to quantify aging saliency in LCRs, highlighting the craniofacial, dental, and cervical spine regions as significant areas for age-related changes [42]. Seo et al. compared six CNN models for CVM classification, with Inception-ResNet-v2 performing best, achieving over 90% accuracy [39]. Kim et al. developed a stepwise segmentation-based model focusing on C2–C4 regions, improving CVM estimation accuracy [43]. Mohammed et al. utilized CNNs for multiclass classification of skeletal growth maturation, reporting high accuracy in predicting CVM stages and gender [44]. These studies underscore the potential of deep learning methods in capturing complex patterns associated with vertebral maturation and age estimation without relying on manually defined landmarks.

Despite these advancements, current methods face several challenges. Many existing approaches focus primarily on specific age groups, particularly children and adolescents undergoing active growth, with limited applicability to adult populations. Additionally, there remains a need for comprehensive evaluation of different vertebral regions and their relative contributions to age estimation accuracy.

To address these gaps, this study introduces a comprehensive framework for automated age estimation based on cervical vertebrae regions in lateral cephalometric radiographs. Unlike previous studies focused primarily on children or specific age groups, our approach is designed to capture both developmental and degenerative aging features across a wide age spectrum. Specifically, we employ mask segmentation to control input regions and systematically evaluate the contribution of different vertebral structures and surrounding tissues across multiple input modes. Furthermore, we quantify and visualize the most critical regions for age estimation across different age groups. This study establishes a robust, multi-tissue analytical baseline for cervical vertebrae-based age estimation and provides novel methodological insights for applications in both orthodontics and forensics.

## 2. Results

### 2.1. The MAE of Age Estimation for Each Input Mode

In this study, the performance of age estimation for the four input modes: cervical vertebrae contour (C), cervical vertebrae mask (M), cervical vertebrae image (CV), and cervical spine region (SR) divided by fixed coordinates as proposed in ARDA, as defined in the Methods section, was quantified using the Mean Absolute Error (MAE). The MAE values for individual vertebrae and their combinations across all modes and age groups are presented in Table 1.

For the contour and mask modes, the MAEs of all 7 combinations of cervical vertebra regions in the 4–25 years age group are less than 4.5 years, and the MAEs of the 30–40 years age group are greater than 10 years. In addition, for both the cervical vertebra and cervical spine region modes, the MAEs for most of the combinations are less than 10 years within the range of 4–40 years (except for the C1–2 cervical region in the 36–40 years age group in the cervical vertebra mode). The overall MAE of the input combination C1–C4 in the cervical spine region mode is the lowest among the four input modes. Here, C1–C4 denotes the combined analysis of all four cervical vertebrae (C1, C2, C3, and C4). The contour and mask modes have similar MAEs across input combinations and the poorest overall performance among the four input modes. The age estimation performance exhibits a similar distribution for all four input modes. Specifically, the accuracy of the combined cervical spine is superior to that of the individual cervical vertebrae, and the accuracy of the continuous combined cervical spine combinations (e.g., C1–2 + C3, C3 + C4) surpasses that of the discontinuous combined cervical spine combination (e.g., C1–2 + C4). The estimation accuracy is highest for the C1–C4 combination for each input mode compared with the other cervical region combinations. The overall MAE across all age groups is lowest for the cervical spine region input mode and the C1–C4 combination, which contains the richest image information.

Before age 25, the MAEs for the age estimates at C1–2, C3, and C4 are less than 5 years for all the input modes. For the 26–40 years age group, the MAEs for the contour and mask modes are significantly greater than those for the cervical vertebra and cervical spine region modes for all the input combinations. C3 and C4 have been commonly utilized in prior studies on age estimation from the cervical spine. Our examination of the four input modes reveals that the estimated accuracies of C3 and C4 were comparable, with an MAE difference of approximately 10%. C1–2 has been infrequently used in prior research because of its intricate architecture, which presents challenges for precise measurement. The results of this study reveal that the MAE of C1–2 for age estimation across the four input modes is not greater than 4.21 years for subjects younger than 25 years.

### 2.2. The ARDA Map of the Cervical Vertebrae Region from the Ages of 4 to 40 Years

**Figure 1:**
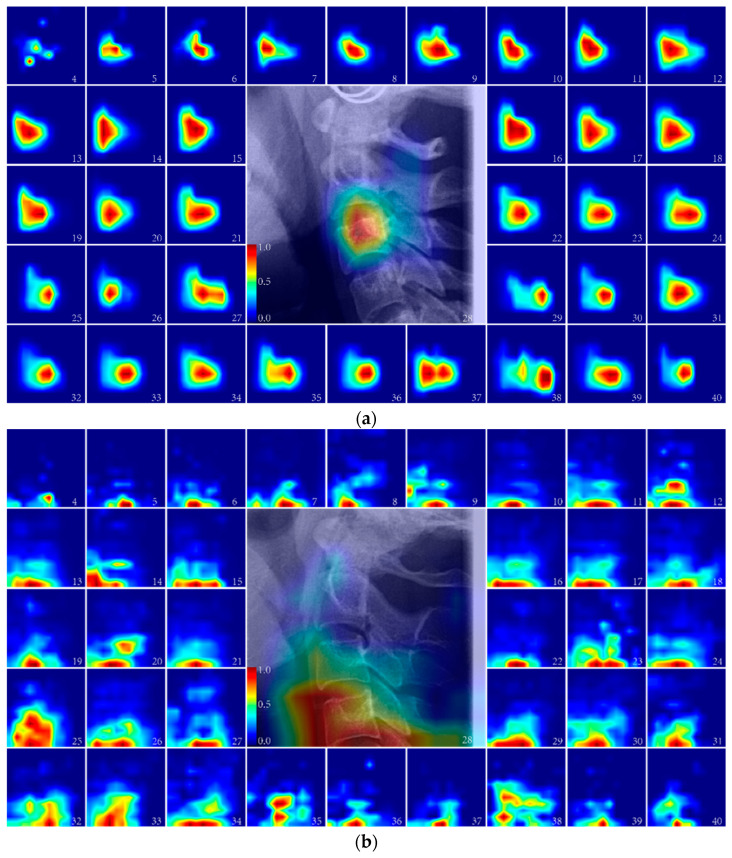

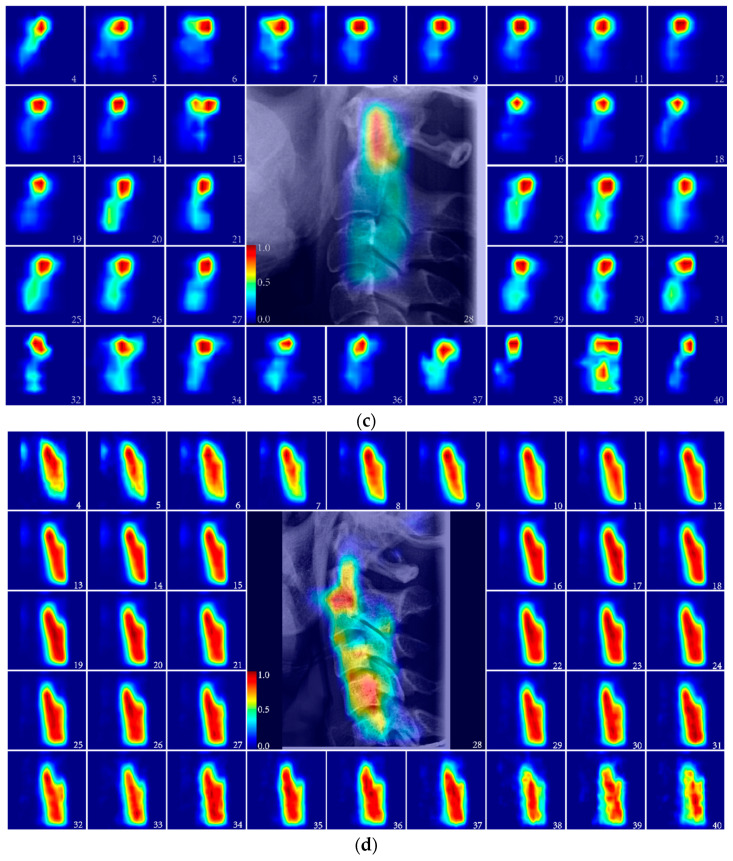
The ARDA map of each input mode from the ages of 4 to 40 years old. The central panel displays the cervical vertebrae region of a randomly selected 28-year-old subject overlaid with its corresponding ageing salience map to show the relative position between the ARDA map and the cervical spine region of the LCR image. (**a**) The ARDA map of C mode; (**b**) The ARDA map of M mode; (**c**) The ARDA map of CV mode; (**d**) The ARDA map of SR mode.

Since the four input modes of cervical vertebrae region C1–C4, have the highest estimation accuracy and contain the most age information compared to the other cervical vertebrae range, C1–C4 can better show the age-related significance region. Therefore, the ARDA maps of C1–C4 for these four input modes were derived to understand the age-related significance region for each input mode (Figure 1).

It can be seen that the age-related region of C mode is mainly in the C3 (Figure 1a). The strongest correlation in the age-related regions of M mode is in the C4, while C3 also has a strong correlation (Figure 1b). For the age-related regions of cervical vertebra mode, C1–2 has the strongest correlation before the age of 18, while C3 and C4 have a weak correlation. However, after the age of 18, C1–2 has the strongest correlation, and C3 and C4 also have a strong correlation (Figure 1c). The age-related regional correlation C1–C4 of the SR model is strong, and there is no significant difference in the intensity of age-related correlation (Figure 1d). The general trend is that the range of age-related regions gradually expands with the increase of input information.

## 3. Discussion

In this study, we find that the mask and contour input modes, which rely solely on osseous morphology, demonstrated substantially inferior performance compared to the regional mode integrating multiple vertebrae and surrounding soft tissues. This finding challenges the conventional paradigm in forensic anthropology that primarily depends on skeletal morphology for age estimation. Notably, in individuals over 25 years of age, methods relying exclusively on bony structures resulted in estimation errors exceeding 10 years, highlighting the fundamental limitations of morphological features alone for adult age estimation.

In contrast to traditional approaches that depend on manual measurements of single vertebrae [6,21], this deep learning framework reveals more complex biological aging mechanisms. Although previous studies achieved limited success using single vertebrae such as C3 and C4 in adolescent cohorts [45,46,47,48,49], the present study demonstrates a marked decline in the efficacy of these methods for adult age estimation [38,50]. This age-related performance disparity stems from distinct biological aging processes dominating different life stages: skeletal growth changes prevail during adolescence, whereas degenerative changes in soft tissues become predominant in adulthood.

To elucidate the relationship between imaging features and age, we employed Grad-CAM-generated aging saliency maps for visual analysis. These maps revealed that age-related salient regions vary across input modes (e.g., the contour mode primarily highlighting C3, while the mask mode showed the strongest correlation in C4) and exhibit dynamic changes with age (e.g., increased saliency in C1–2 after age 18). This approach not only enhances the interpretability of the prediction mechanism but also provides a quantitative basis for understanding cervical spine aging across different age groups (Figure 1).

The consistent performance hierarchy across input modes (SR > CV > M ≈ C) underscores the importance of integrating multi-tissue information and anatomical continuity. The combination of soft tissues (e.g., intervertebral discs and ligaments) with bony structures proved to be critically important. We hypothesize that the key features captured by the SR mode may correspond to well-established, age-related degenerative pathophysiological changes—such as disc dehydration, ligamentous calcification, and reductions in intervertebral space height—which manifest as alterations in texture, density, and spacing on radiographs. These features provide more continuous and sensitive biological markers of aging compared to relatively static bony morphological characteristics. The superior performance of continuous vertebral combinations (e.g., C1–2 + C3) over discontinuous combinations (e.g., C1–2 + C4) further suggests that anatomical continuity may reflect synergistic aging mechanisms. The significant enhancement in predictive accuracy when bony features are analyzed within a continuous anatomical context including soft tissues underscores the limitations of isolated feature analysis and emphasizes the superiority of a multi-tissue integrative analytical paradigm.

As summarized in Table 2 below, previous studies can be broadly categorized into two groups: (1) traditional methods relying on manual staging or handcrafted morphological measurements of specific vertebrae (e.g., C2, C3, C4), which are effective primarily for assessing skeletal growth during adolescence; and (2) recent deep learning approaches that automate the analysis, yet most remain focused on adolescent cohorts or specific vertebral bodies, often neglecting the surrounding soft tissues and the continuous nature of aging beyond growth completion.

In this study, the soft tissue-based regional input mode (SR) embodies this paradigm shift by offering distinct advantages over discrete staging methods such as Cervical Vertebral Maturation (CVM). Specifically, this framework generates continuous and quantifiable age estimates rather than discrete staging outcomes, thereby enabling higher-resolution age inference. Furthermore, the model explicitly leverages degenerative information from peri-vertebral soft tissues—a dimension entirely absent in traditional morphology-based analytical systems. Additionally, the method achieves full-process automation and data-driven analysis, not only effectively reducing the subjectivity of manual assessment but also extracting complex nonlinear patterns from imaging data that are imperceptible to the human eye, such as textural changes in intervertebral discs and ligaments.

The reliable benchmark and methodological framework established in this study offer a novel technical pathway for addressing the challenge of age estimation in fragmented skeletal remains. Even with incomplete vertebral samples, the use of different vertebral input modes and combinations may still yield reliable age estimates, which holds significant potential for the identification of unknown individuals in forensic contexts. In orthodontics, incorporating soft tissue evaluation is expected to significantly improve the accuracy of growth potential prediction, particularly in cases with abnormal development patterns.

Notably, age estimation accuracy declined for older adults, with the 36–40 year cohort showing an MAE of 5.43 years even using the optimal SR mode. This reflects a key challenge in adult age estimation: after skeletal maturation, aging manifests primarily through subtle degenerative changes in intervertebral discs, ligaments, and bone density—features that are inherently limited in contrast and resolution on 2D lateral cephalograms. Future studies incorporating 3D imaging modalities such as CT for bony architecture and density and MRI for disc and ligament assessment, which could provide richer degenerative biomarkers to improve estimation in older populations.

Despite these advancements, several limitations must be acknowledged. The dataset was sourced from a single hospital in Northwest China, which may limit generalizability to other populations and necessitate recalibration or retraining for different ethnic groups. Additionally, our analysis was limited to vertebrae C1 through C4 due to the standard imaging protocol for lateral cephalometric radiographs, which includes thyroid shielding and typically precludes visualization of the lower cervical vertebrae (C5–C7). This constraint may lead to the omission of potentially valuable age-related information, as the lower cervical segments often exhibit earlier and more pronounced degenerative changes in adulthood. Nevertheless, the robust performance achieved using the consistently captured C1–C4 vertebrae in routine LCR imaging demonstrates the practical utility of the proposed framework. Future research could further validate and extend this method in datasets where full cervical spine imaging is available. Moreover, the age range (4–40 years) precludes application to older populations and the study of advanced aging processes. Furthermore, manual segmentation of vertebral regions may introduce some subjectivity.

While acknowledging these constraints, the multi-tissue integrative analysis framework proposed in this study establishes a more robust benchmark for cervical spine-based age estimation. Future work will expand the dataset to include more diverse populations and broader age ranges, and develop dedicated network architectures capable of explicitly modeling the synergistic degenerative relationship between bony and soft tissues. This will further validate and promote the proposed paradigm, providing continued insights for both orthodontic practice and forensic applications.

## 4. Materials and Methods

**Figure 2:**
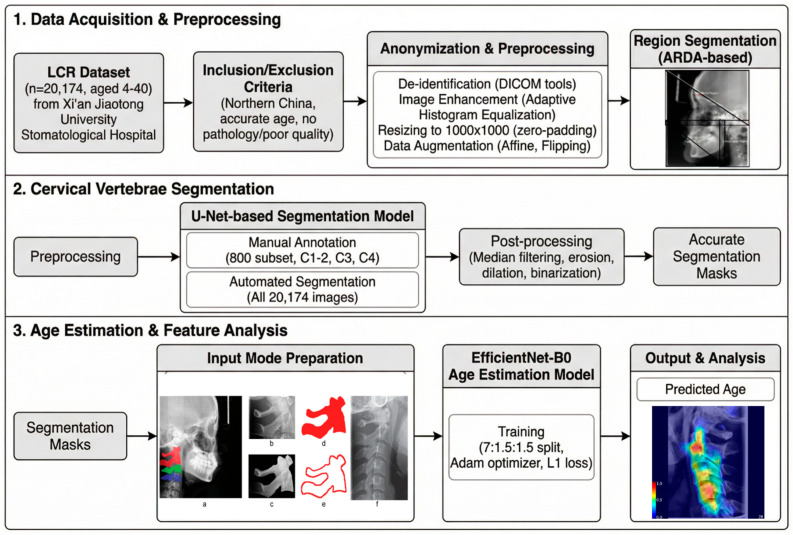
Workflow for cervical vertebrae age estimation from LCRs.

The study is designed for cervical vertebrae age estimation from LCRs. The workflow is as Figure 2. First, in the Data Acquisition and Preprocessing stage, LCR images were collected and standardized. This involved anonymization, contrast enhancement via adaptive histogram equalization, and resizing to a uniform resolution with aspect-ratio-preserving padding. Second, the Cervical Vertebrae Segmentation stage employed a U-Net-based model to generate precise masks for the vertebrae C1–C4. The model was trained on expert-annotated data and applied fully automatically to isolate individual vertebrae and their surrounding regions. Third, in the Age Estimation & Feature Analysis stage, a deep learning framework based on EfficientNet-B0 was implemented. Four distinct input modes—Contour (C), Mask (M), Cervical Vertebrae (CV), and Cervical Spine Region (SR)—were systematically evaluated to assess the contribution of different tissue types and anatomical contexts. Model training utilized the Adam optimizer with exponential learning rate decay, and performance was quantified using Mean Absolute Error (MAE). Furthermore, gradient-weighted class activation mapping (Grad-CAM) was applied to visualize age-related saliency, providing interpretable insights into the regional importance across age groups. This integrated workflow ensures a robust, reproducible, and clinically interpretable approach to cervical spine-based age assessment.

### 4.1. Study Population

**Figure 3:**
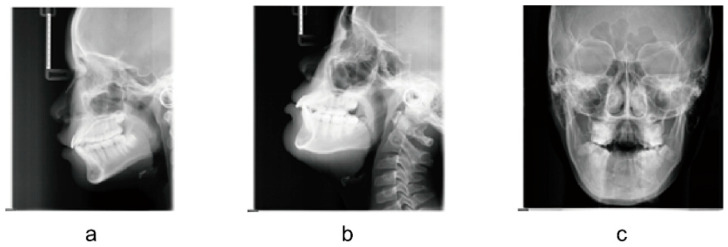
Representative examples of unqualified images. (**a**) Incomplete LCR image. (**b**) Incorrect imaging posture. (**c**) Wrong imaging position.

**Figure 4:**
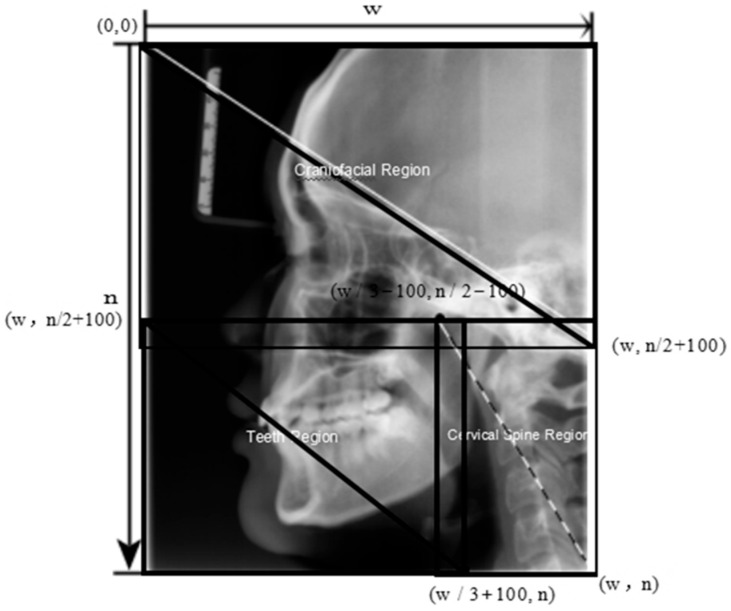
Position of the tooth, craniofacial, and cervical regions in an LCR image. The top-left and bottom-right coordinates are specified for each area. With the upper left corner of the LCR image as the origin of the coordinate system, the X-axis positive direction is to the right, and the Y-axis positive direction is downwards. W is the width, and n is the height.

The LCR dataset containing 20,174 patients aged 4–40 years was obtained from the database of Xi’an Jiaotong University Stomatological Hospital in the northwest region of China. This study was approved by the Affiliated Stomatological Hospital of Xi’an Jiaotong University Health Science Center (Approval number: xjkqll [2022] NO.30). All patients provided written informed consent before undergoing LCR imaging, acknowledging that their images could be used for scientific research. As a retrospective study, all images were originally acquired for diagnostic or therapeutic purposes and did not involve additional radiation exposure. The age of each subject was calculated based on the date of birth documented in their official national identity card, which was used during patient registration. This was achieved by subtracting the imaging date from the date of birth, dividing by 365.25 (to account for leap years), and rounding to the nearest hundredth. The inclusion criteria for the LCR dataset were as follows: (1) accurate age records and (2) the northern region of China. The final composition and size distribution of the resulting dataset across different age groups are detailed in Table 3. The exclusion criteria were as follows: (1) incorrect LCR imaging position; (2) images with poor image quality; (3) head, face, and neck tumours or diseases associated with bone and tooth integrity destruction. Examples of unqualified images are shown in Figure 3; (4) diseases related to bone maturation (e.g., osteoporosis, endocrine disorders) and medications (e.g., hormone-based drugs).

### 4.2. Age Group Stratification

The age range from 4 to 40 years was chosen in this study because it could basically cover the development period and ageing period based on lateral radiograph age estimation. Children under the age of 4 years are rarely examined with lateral radiographs, and X-ray examination cannot be performed on research subjects for research purposes due to medical ethical reasons, so we chose the age of 4 years as the lower limit of our study age range.

The study population was stratified into two age groups (4–25 years and 26–40 years) based on established biological milestones and empirical observations from pilot analyses. The age of 25 years marks the completion of skeletal maturation, including fusion of cervical vertebral epiphyseal rings, after which degenerative changes (e.g., intervertebral disc degeneration, bone density loss) become predominant [26,27,28,29,30,31,51]. This transition was further supported by our preliminary deep learning-based feature analysis, which indicated distinct aging patterns in cervical spine regions before and after approximately 25 years of age. The 26–40-year group was specifically included to explore degenerative changes using deep learning, which can capture subtle features not readily quantified by conventional methods [42]. All subjects were divided into 7 groups according to 5-year age intervals. The age and sex distributions of the subjects are shown in Table 4.

### 4.3. Data Anonymization and Preprocessing

A dedicated medical imaging anonymization tool was utilized in this study to perform standardized de-identification on all data in Digital Imaging and Communications in Medicine (DICOM) format. This procedure systematically removes embedded personally identifiable information from the image files while retaining only age and gender as non-identifiable analytical variables required for the research, thereby ensuring comprehensive protection of patient privacy. All imaging data underwent complete anonymization to eliminate any risk of personal information exposure. The age of patients at the time of imaging was accurately determined by matching unique identification numbers with corresponding birthdates retrieved from the Hospital Information System (HIS).

All LCRs were processed through a standardized preprocessing workflow. A computer vision library was employed for image enhancement and normalization, beginning with adaptive histogram equalization to optimize contrast, followed by resizing to a uniform resolution of 1000 × 1000 pixels. Aspect ratio preservation was maintained through zero-padding with black borders to achieve dimensional standardization. During the model training phase, a multimodal data augmentation strategy was implemented on the training dataset to improve model generalization, incorporating random affine transformations along with random horizontal and vertical flipping.

### 4.4. The Cervical Vertebrae Region Segmentation

The ageing-related dynamic attention (ARDA) method proposed in a previous study [42] provides the average ageing significance of bone and dental tissue in different areas of the head and neck. Using the ARDA distribution, each LCR image in the dataset was divided into three parts: the tooth region (TR), craniofacial region (CR), and cervical spine region (SR). The position of each region in the LCR image is constant. In this study, we used the same division of the cervical spine region. The LCR image was segmented into three sections based on pixel location (Figure 4).

**Figure 5:**
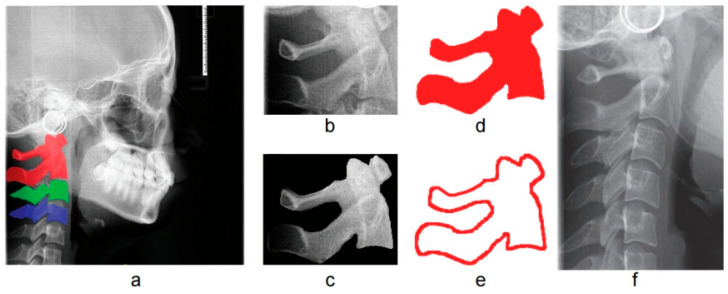
Different input modes for cervical vertebrae age estimation in the study design (using C1–2 as an example) (**a**) Individual cervical vertebrae (C1–2, C3, C4) in LCR images and their segmentation masks. (**b**) C1–2 region defined by the minimum upper and lower boundaries of the segmentation masks ($I^{reg}$). (**c**) C1–2 vertebral body and spinous process ($I^{seg}$). (**d**) Mask of the cervical C1–2 region ($I^{mask}$). (**e**) Contour template of the segmented cervical C1–2 region ($I^{con}$). (**f**) Cervical region divided into fixed coordinates as proposed in ARDA ($I^{ARDA}$).

As shown in Figure 5a, C1–C4 were included in most of the LCR images. C5 was included in only a few LCR images due to the hindrance to the thyroid protection collar during radiation exposure, so this study focused on C1–C4 and its surrounding tissues. For anatomical reasons, C1 and C2 overlapped on the LCR image and were therefore treated as a single cervical vertebra, C1–2, in this study.

### 4.5. Segmentation Masks for C1–2, C3, and C4

To obtain accurate segmentation masks for cervical vertebrae, we employed a U-Net-based deep learning model in a fully automated pipeline. The model was developed through a two-stage process to ensure reliability and reproducibility.

Firstly, to train and validate the segmentation model, a subset of 800 lateral cephalometric radiographs was randomly selected. This subset was independently annotated by two board-certified orthodontists, each with over five years of clinical experience. Using ITK-SNAP software (Insight Toolkit—Snake Automatic Partitioning, Version 3.80; University of Pennsylvania, Philadelphia, PA, USA), the annotators manually delineated the contours of the C1–2 (considered as a combined structure due to frequent anatomical overlap), C3, and C4 vertebral bodies, including the posterior arches where clearly visible.

The segmentation mask acquisition module uses the X-ray cephalometric lateral image as input to a U-Net-based segmentation network, producing an image with segmentation results. This process is fully automated to segment the vertebrae in all 20,174 images within the main dataset. No manual intervention or correction was performed at this stage. The segmentation mask undergoes median filtering, erosion, dilation, binarization, and a final dilation step to obtain an accurate cervical vertebrae segmentation mask.

### 4.6. Input Conditions

Cervical vertebrae can aid in age estimation, but forensic scenarios may involve incomplete information, such as shredded bodies or skeletal remains, and the cervical vertebrae may be absent. Considering the challenges encountered in forensic practice, we also designed various combinations for each input mode, including C1–2, C3, C4, C3+C4, C1–2+C3, C1–2+C4, and C1–C4, where C1–C4 denotes the combined analysis of all four cervical vertebrae (C1, C2, C3, and C4).

As shown in Figure 5, we designed four input modes to evaluate the performance of age estimation in various scenarios with different information richness. Specifically, these four inputs include: (1) cervical vertebra region defined by the minimum boundaries of the segmentation mask ($I^{reg}$, Figure 5b), (2) cervical vertebrae with surrounding tissue removed ($I^{seg}$, Figure 5c), (3) cervical vertebrae segmentation mask ($I^{mask}$, Figure 5d), (4) cervical vertebrae segmentation contour template ($I^{con}$ Figure 5e). For reference, the cervical vertebrae region delineated by the fixed coordinates proposed in ARDA ($I^{ARDA}$) is shown in Figure 5f.

### 4.7. Ageing Feature Extraction

Compared to other CNNs, EfficientNet-B0 achieves optimal performance with the fewest parameters, making it the baseline age estimation model and aging feature extractor. The comparison of age estimation performance and parameter count of CNN models that perform well on natural images has been demonstrated in our previous study [42]. In this study, the EfficentNet-B0 model is used for age estimation for different types of inputs in the cervical region. The EfficientNet-B0 architecture is provided in Table 5.

The LCR dataset was divided into training, validation, and test sets in a 7:1.5:1.5 ratio for training the age estimation and gender classification models. Based on the classification models trained on the training, validation, and test sets, age estimation was performed using single cervical vertebrae and multiple cervical vertebrae combinations, with the cervical vertebrae regions guided by ARDA, for each input condition. The corresponding age for the X-ray cephalometric lateral images was then obtained.

The Adam optimizer was employed for model training, with the learning rate dynamically adjusted using an exponential decay strategy. The MAE, a widely used metric for the age estimation task, was used to evaluate the performance of the model in this study. Age-related cervical changes are a continuous variable, and age estimation of the cervical vertebrae using deep learning is a regression task. Therefore, L1 loss was used for model training:

(1) $$L=\frac{1}{N}\sum_{n=1}^{N}\left|y_{n}-y_{n}^{\prime }\right|,$$

where N is the number of samples, the $y_{n}$ and $y_{n}^{\prime }$ are the age and predicted age of the n-th LCR image, respectively.

Four distinct input modes were designed to evaluate the contribution of different information types to age estimation performance: (1) Cervical vertebrae contour (C): the binary contour outline of the vertebrae; (2) Cervical vertebrae mask (M): the binary segmentation mask of the vertebrae; (3) Cervical vertebrae image (CV): the pixel values within the segmentation mask, isolating the vertebrae from surrounding tissues; and (4) Cervical spine region (SR): a fixed rectangular region around the vertebrae (as defined in ARDA [42]), containing both vertebral and surrounding soft tissue information.

### 4.8. The Saliency Map Generated via Grad-CAM

In this study, the saliency of development and ageing was obtained by the saliency map generated via Grad-CAM [42]. In this method, the gradients of the neural network output results with respect to the feature maps are used as weights, from which the weighted sum of the feature maps is calculated and the saliency maps of the input images with respect to the output results are obtained.

To quantify age-related changes, we introduced the concept of “Ageing salience”—a quantitative measure representing the correlation between specific regions in LCR images and the aging process. This metric allows intuitive visualization of which anatomical regions exhibit the most significant age-related changes. The visual representation of these ageing salience values through Grad-CAM methodology is termed “Ageing salience map”, where warmer colors (closer to red) indicate regions with stronger correlation to aging processes.

First, the weight of k-th channel of the feature map is calculated from the gradient of the output result w.r.t the feature map:

(2) $$\alpha_{k}=\frac{1}{Z}\sum_{i}\sum_{j}\frac{\partial y^{\prime }}{\partial F_{\left(i,j\right)}^{k}},$$

where $F_{ij}^{k}$ is the feature variable at position (i,j) in the k-th channel of the feature map, $y^{\prime }$ is the predicted age, and Z is the number of feature variables in the k-th channel. The saliency map s is obtained by the weighted sum of the feature map channels:

(3) $$s=ReLU(\sum_{k}\alpha_{k}F^{k}),$$

where ReLU(⋅) is the ReLU function used to filter out regions with negative values, which is defined as follows:

(4) $$ReLU\left(x\right)=\{\begin{array}{c} x\;\;\;\;\;\;\;if\;x>0\\ 0\;\;\;\;\;otherwise \end{array}.$$

For comparison with the tissue in the input image, s is resized to the dimensions of the input image by interpolation.

To extend this from individual-level to population-level analysis, we developed the Aging Related Dynamic Attention (ARDA) mechanism, which captures dynamic, age-specific attention patterns across vertebral regions while eliminating individual variations among subjects of the same age.

It is necessary to represent age-related salience at the age scale rather than at the individual scale to analyze the distribution of developmental and ageing salience in cervical spine regions at different ages and their dynamic patterns. The mean of the saliency maps of all samples of the same age can be used as the general saliency map of this age, since the position of the patient in the LCR image is basically fixed. Development and ageing saliency map $S^{a}$ corresponding to age a are generated as follows:

(5) $$S_{\left(i,j\right)}^{a}=\frac{1}{N_{a}}\sum_{n=1}^{N_{a}}s_{\left(i,j\right)}^{n},$$

where the subscript ij indicates that the position of the saliency value in the saliency map is (i,j).

## Figures and Tables

**Table 1:** MAE of age estimation for four input modes of cervical vertebrae.

Input Modes	Cervical Vertebrae	4–10	11–15	16–20	21–25	26–30	31–35	36–40	4–25	26–40	All
C	C1–2	1.57	2.51	2.91	4.05	6.88	11.08	15.66	2.74	8.93	3.43
	C3	1.68	2.18	2.76	4.23	7.01	12.26	17.23	2.60	9.49	3.37
	C4	1.52	2.01	2.52	3.94	7.51	11.89	17.33	2.39	9.72	3.21
	C3 + C4	1.32	1.96	2.81	3.63	5.88	10.84	14.67	2.37	8.13	3.01
	C1–2 + C3	1.29	2.00	2.70	3.66	5.98	10.71	13.84	2.35	8.06	2.99
	C1–2 + C4	1.39	1.81	2.52	3.68	6.30	10.47	16.05	2.25	8.44	2.94
	C1–C4	1.18	1.67	2.56	3.53	5.51	10.09	14.13	2.15	7.64	2.75
M	C1–2	1.61	2.41	2.56	4.21	7.37	12.13	17.37	2.63	9.69	3.42
	C3	1.42	2.09	2.62	4.38	7.35	12.65	17.16	2.52	9.79	3.32
	C4	1.46	2.23	2.73	3.70	6.39	11.31	15.36	2.49	8.65	3.18
	C3 + C4	1.35	1.80	2.65	3.53	6.00	11.58	16.25	2.25	8.56	2.95
	C1–2 + C3	1.22	1.79	2.59	3.94	6.59	11.32	14.66	2.28	8.69	2.99
	C1–2 + C4	1.09	1.96	2.90	3.54	5.30	9.67	14.90	2.34	7.48	2.91
	C1–C4	1.44	1.69	2.57	3.43	5.54	9.46	13.46	2.18	7.42	2.76
CV	C1–2	1.22	1.68	2.40	3.18	4.64	8.29	10.57	2.05	6.23	2.52
	C3	1.22	1.27	2.14	2.77	4.05	6.83	9.69	1.74	5.38	2.15
	C4	1.12	1.41	2.19	2.68	3.95	6.76	9.30	1.78	5.26	2.17
	C1–2 + C3	0.98	1.24	2.02	2.75	3.49	6.57	8.94	1.66	4.88	2.02
	C1–2 + C4	1.06	1.32	2.12	2.49	3.66	6.68	9.07	1.69	5.04	2.06
	C3 + C4	1.14	1.15	1.94	2.60	3.93	6.31	8.58	1.60	5.05	1.98
	C1–C4	1.06	1.16	1.84	2.41	3.30	5.73	8.49	1.54	4.50	1.86
SR	C1–2	1.08	1.17	1.80	2.52	3.65	6.98	8.70	1.55	5.06	1.94
	C3	1.24	1.11	1.96	2.77	4.07	6.91	9.11	1.63	5.36	2.04
	C4	1.15	1.04	1.70	2.28	3.53	6.77	8.45	1.44	4.90	1.82
	C1–2 + C3	0.96	1.06	1.67	2.20	3.22	5.60	7.45	1.40	4.30	1.72
	C1–2 + C4	1.05	1.33	2.00	2.62	3.68	6.74	9.17	1.68	5.07	2.06
	C3 + C4	1.05	1.11	1.82	2.28	2.66	5.26	7.12	1.49	3.82	1.75
	C1–C4	0.96	0.83	1.48	1.91	2.36	4.06	5.43	1.20	3.14	1.41

**Table 2:** Comparison of methodologies for cervical vertebrae-based age estimation.

	Traditional Staging & Morphometric Methods	Prior Deep Learning Studies	This Study
Primary Target Age Group	Predominantly adolescents	Primarily adolescents and young adults (typically ≤25 years)	Broad spectrum: 4–40 years (encompassing both growth and early degenerative phases)
Input Information	Manual assessment of bony morphology (e.g., concavity, shape, ratios of C2–C4)	Automated analysis of bony structures (e.g., segmented C2–C4 vertebrae or full LCR images)	Multi-tissue integration: Systematic evaluation of four input modes, with superior performance from the Soft-tissue Region (SR) mode combining bony and surrounding soft tissues.
Output Type	Discrete stage (e.g., CVM Stage I-VI)	Discrete stage (CVM) or continuous age (for narrow age ranges)	Continuous age estimate across the entire lifespan studied.
Analytical Strength	Established clinical correlation with growth peaks.	Automation, objectivity, and the ability to model complex patterns in bone.	1. Paradigm shift: First to systematically quantify the critical contribution of peri-vertebral soft tissues to age estimation, especially in adults (>25 years). 2. Design validation: Comprehensive ablation via four input modes (C, M, CV, SR) and multiple vertebral combinations, providing empirical evidence for the importance of anatomical continuity and multi-context integration. 3. Explainability: Use of Grad-CAM saliency maps provides interpretable, age-specific biological insights into feature importance across tissues.
Major Limitation	Highly subjective, unsuitable for adults, cannot capture degenerative changes.	Often limited to bone-only analysis, lack of interpretability regarding tissue contribution, limited validation on adults	

**Table 3:** The size distribution of LCR image dataset used in our study.

Width	Height		Number
1804	2136		5338
2136	2304		2050
2140	2304		4000
2144	2304		7179
2148	2304		1524
Others		83	
Sum		20,174	

**Table 4:** Age and sex distributions of the LCR dataset.

Age Group (Year)	Sex		Subset			Total
	Male	Female	Training Set	Validation Set	Test Set	
4–10	1264	1335	1822	393	384	2599
11–15	3072	4580	5354	1148	1150	7652
16–20	1713	2878	3211	690	690	4591
21–25	815	2229	2137	454	453	3044
26–30	297	1155	1020	210	222	1452
31–35	102	475	408	86	83	577
36–40	39	220	190	37	32	259
Total	7302	12,872	14,142	3018	3014	20,174

**Table 5:** EfficientNet-B0 network architecture.

Stage	Operator	Resolution	Channel	Layers
I	${\hat{F}}_{i}$	${\hat{w}}_{i}$ × ${\hat{n}}_{i}$	${\hat{C}}_{i}$	${\hat{L}}_{i}$
1	Conv3 × 3	1000 × 1000	32	1
2	MBConv1, k3 × 3	500 × 500	16	1
3	MBConv6, k3 × 3	500 × 500	24	2
4	MBConv6, k5 × 5	250 × 250	24	2
5	MBConv6, k3 × 3	125 × 125	80	3
6	MBConv6, k5 × 5	62 × 62	112	3
7	MBConv6, k5 × 5	62 × 62	192	4
8	MBConv6, k3 × 3	31 × 31	320	1
9	Conv1 × 1 & Pooling & FC	31 × 31	1280	1

## Data Availability

The author declares that data supporting the results of this study can be found at https://pan.baidu.com/s/1ltPPdrSjU3nijBMUAzYNrw (accessed on 10 November 2025). The code used in this study was made fully publicly available at https://github.com/LiuNingtao/ARDA (accessed on 10 November 2025).
